# The Concurrent Criterion Validity of the 32-Item Fitness-to-Drive Screening Measure

**DOI:** 10.3389/fpsyg.2019.00253

**Published:** 2019-02-14

**Authors:** Shabnam Medhizadah, Sherrilene Classen, Andrew M. Johnson

**Affiliations:** ^1^University of Florida, Gainesville, FL, United States; ^2^University of Western Ontario, London, ON, Canada

**Keywords:** psychometric, automobile driving, proxy raters, ROC (Receiver Operating Characteristic) curve, sensitivity and specificity (MeSH)

## Abstract

**Background:** The Fitness-to-Drive Screening Measure^©^ (FTDS) is a free online screening tool that identifies at-risk older drivers. This tool screens for at-risk drivers using proxy rater responses (family, friends, and caregivers) to 54 driving-related items. Consumer usage analysis of the FTDS determined that reducing the time commitment to complete the 54-item FTDS might increase usability and uptake of the tool. To address this need, we used classical test theory and exploratory factor analysis to construct a 32-item version of the FTDS. This study aims to establish the concurrent criterion validity of the 32-item FTDS.

**Method:** Two hundred older driver on-road assessments and Two hundred caregiver FTDS responses were used to generate a receiver operating characteristic (ROC) curve, in which we plotted the rate of true positives against the rate of false positives, calculated the area under the curve (AUC), and used Youden's index to identify the optimal cut-point for the 32-item FTDS. In this study, the true positive rate was the 32-item FTDS' ability to predict a fail when the older driver actually failed the on-road assessment, and the false positive rate was the the 32-item FTDS' ability to predict a pass when the older driver actually passed the on-road assessment. We computed the sensitivity, specificity, positive predictive value, negative predictive value and total number of misclassifications for the optimal cut-point.

**Results:** The ROC curve results indicated an acceptable AUC, with a magnitude of 0.75, *p* < 0.05. At the optimal cut-point of 4.87, the 32-item FTDS had a sensitivity of 0.74, specificity of 0.69, positive predictive value of 0.30, negative predictive value of 0.93 and 61 (of 200) misclassifications.

**Conclusion:** Although the 32-item FTDS met the criterion (AUC 0.75, *p* < 0.05.) for good concurrent criterion validity in predicting older driver on-road outcomes, it also misclassified 30% of the drivers and as such may be overly sensitive.

## Background

The Fitness-to-Drive Screening Measure^©^ (FTDS) is a reliable and valid tool for identifying at-risk older drivers (Classen et al., 2015). Using Google Analytics reports, Classen et al. (2016) found that numerous users are accessing the FTDS, but not completing it in its entirety which may potentially impact the measure's uptake. A shorter FTDS has been proposed and constructed to overcome the lengthy (and potentially problematic) completion time. The psychometric properties of the 32-item measure look promising as it correlates well with the original FTDS measure (Medhizadah et al., 2018). Establishing the validity of the 32-item FTDS will be the first step toward empirically establishing the measure's potential to differentiate between older drivers who would pass or fail an on-road assessment.

### Fitness-to-Drive Screening Measure^©^

The Fitness-to-Drive Screening Measure (previously called the Safe Driving Behavior Measure) is a free online screening measure, available at www.fitnesstodrivescreening.com. The 54-item FTDS was developed and validated to address the growing importance of identifying at-risk older drivers (Classen, 2015). The measure was developed to be a community-based screening tool that is accessible, relevant, culturally sensitive, and geographically representative, with utility for older drivers and concerned proxy raters (e.g., formal/informal caregivers, family members or friends; Classen et al., 2010). This measure enables proxy raters who have driven with the individual in the last 3 months, to identify at-risk older drivers (≥65 years of age). The web-based FTDS takes approximately 20 min to complete and consists of three sections. Sections A and B consist of demographic questions about first the driver and then the proxy rater. Section C asks the proxy rater to use his or her observations of the driver to provide judgments on 54 driving-related items. The 54 items are rated using a Likert scale: 1 = *very difficult*, 2 = *somewhat difficult*, 3 = *a little difficult*, 4 = *not difficult*. The driving skills assessed by the FTDS items range from easy (e.g., item 4; how difficult is it for the driver to check car mirrors when changing lanes?) to challenging (e.g., item 50; how difficult is it for the driver to turn left across multiple lanes when there is no traffic signal?). Per Classen (2015), responses in this section of the FTDS are used to classify the driver as an *at-risk driver* (although the driver can perform some basic driving skills, there are immediate safety concerns that must be addressed), *routine driver* (some driving skills are causing concern, and the driver is showing early signs of needing intervention), or *accomplished driver* (overall the driver does not exhibit insufficiency in their driving skills but may experience some driving difficulty in challenging situations). Subsequently, the proxy rater is provided with recommendations (including a key form that highlights overall areas of driving difficulty) and resources appropriate for managing the identified level of risk.

Psychometric properties of the 54-item FTDS indicate that the measure is a valid and reliable tool for identifying at-risk older drivers (Classen et al., 2015). Exploratory factor analysis of the FTDS suggested a 2-factor model best represented the constructs of the FTDS. Upon further examination, 14 items were identified as pre-driving items (e.g., open car door) and were removed, resulting in a more homogenous one-factor model. Confirmatory factor analysis results indicated driving evaluators and proxy ratings fit a one-factor model, whereas driver ratings did not. Evaluators and proxy ratings also demonstrated good unidimensionality, but driver ratings did not. Rasch analysis of the rating scale indicated that evaluators, proxies and drivers underused two (cannot do, very difficult) of the five rating categories. Thus, the two rating categories were combined into one rating category: very difficult. Among the three groups of raters, the strongest correlation was between evaluator and proxy ratings (ICC = 0.39, *p* < 0.001). Using proxy ratings, the FTDS demonstrates concurrent criterion validity with the gold standard on-road assessment (area under the curve = 0.72, *p* < 0.001; Classen et al., 2015).

Despite the established reliability, validity and ability to identify at-risk older drivers, many users quit the 54-item FTDS before completing it (Classen et al., 2016). These researchers suggested that decreasing the time needed to complete the measure may potentially increase the utility of the FTDS. Medhizadah et al. (2018) constructed a shorter 32-item FTDS that may decrease completion time and potentially increase utilization of the FTDS as per its original intent. This 32-item FTDS demonstrated excellent factorial validity (as illustrated by an exploratory factor analysis) and internal consistency reliability for each factor (Cronbach's alpha = 0.96, 0.88, 0.88). Medhizadah et al. (2018) noted that the correlation between the 32-item FTDS and the 54-item version of the FTDS was *r* = 0.99.

The 32-item FTDS was developed to be used by proxies to identify at-risk older drivers. These proxy assessments may, however, be used by clinicians (e.g., occupational therapists). Specifically, it may be used to inform clinicians' clinical reasoning when making fitness to drive decisions about the drivers, which can include one of the following outcomes: continued driving, referral to a certified driver rehabilitation specialist, or driving cessation. If the 32-item FTDS is to be used clinically (but completed by proxy raters), then it is necessary to identify a clinically meaningful cutoff score for clinicians, using a currently accepted gold standard for identifying at-risk drivers. Thus, the purpose of this study is to use an existing dataset to establish the concurrent criterion validity of the 32-item FTDS.

## Methods

The University of Florida's Institutional Review Board (IRB201401055) authorized researchers at the University of Western Ontario (UWO), to use de-identified data from the original study, hereafter referred to as the primary study, conducted with community-dwelling licensed older drivers and their proxy raters. The non-medical Research Ethics Board at UWO stipulated that this study was exempt from ethics review (FWA00000121) because this study only had access to and used de-identified data. In the primary study all participants provided written informed consent.

### Participants

In the primary study, community-dwelling, licensed, older drivers (*n* = 200, age = 65–85 years), and their proxy raters (including formal/informal caregivers, family members or friends; *n* = 200, age = 18–65 years) were recruited from communities in North-Central Florida, United States, and Thunder Bay, Ontario, Canada. Drivers were included in the primary study if they: had a valid driver's license, drove at the time of recruitment, and were physically and cognitively able to take part in both the FTDS and on-road assessment. Conversely, drivers were excluded from the primary study if they: had been medically advised not to drive, had experienced seizures, or took medication that impaired their central nervous system. Proxy raters were included in the primary study if they were able to report on the older driver's driving based on observations in the last 3 months. Proxy raters were excluded if they displayed physical or mental conditions that impaired the ability to make valid observations during screening (Classen et al., 2015).

### Measure

#### 32-Item FTDS

The 32-item FTDS is comprised solely of section C items (items determining difficulty for driving behaviors), all of which were scored using the 5-point Likert scale (1 = *cannot do*, 2 = *very difficult*, 3 = *somewhat difficult*, 4 = *a little difficult*, 5 = *not difficult*), measuring the degree of driver ability as observed by the proxy rater. In the primary study, the FTDS was completed by proxy raters before or during the driver's on-road assessment. The measurement used in the analyses presented herein was created by computing a unit-weighted mean of the 32 items on the measure. This composite score was averaged across all the items completed by participants (i.e., missing data was replaced using mean value substitution).

#### On-Road Assessment

In the primary FTDS study (Classen et al., 2015), an occupational therapist who was also a certified driver rehabilitation specialist (Florida site), and a licensed driving school instructor (Ontario site) conducted the on-road assessment. The Florida site consisted of a standardized road course with reliability and validity for older drivers (Classen et al., 2013). The Canadian on-road assessment used a demerit point system consistent with the method used by its licensing authority. The evaluators used a 4-point scale (3 = pass, 2 = pass with restrictions/recommendations, 1 = fail with remediation, 0 = fail) to assess on-road outcomes. Interrater reliability between the evaluators, conducted on the same participants, was 100% (Classen et al., 2010).

For this study dichotomized pass/fail outcomes of the participants' on-road assessments, as assessed by the above-mentioned evaluators, were used for all analyses.

### Data Collection and Management

All the de-identified data were stored on a password-protected server network at UWO and was only accessible to the research team. For the data analysis, older drivers' mean score on the FTDS, the average driver ability on FTDS items as observed by the proxy rater, was obtained by calculating the average of the proxy's responses to the Likert scale items in the measure. For proxy responses with missing data, the sum of proxy's responses was divided by 32 minus the number of items with missing responses. For example, the mean score for a participant missing responses to three items in the 32-item FTDS was averaged based on 29, not 32 items. The lowest possible mean score of driver ability was one (the driver cannot do the driving tasks), and the highest possible mean score was five (the driver has no difficulty completing the driving tasks).

### Analytic Approach

A ROC curve is generated by plotting the rate of true positives (sensitivity) against the rate of false positives (1-specificity). In the present context, *sensitivity* is the screening measure's (32-item FTDS) ability to predict a fail when the older driver actually failed the on-road assessment (Streiner and Cairney, 2007). *Specificity* is the screening measure's (the 32-item FTDS) ability to predict a pass when the older driver actually passed the on-road assessment (Streiner and Cairney, 2007).

The *false positive rate*, also known as a *Type I error*, is when the screening measure predicts a fail, even though the driver actually passed the on-road assessment, and is calculated as 1- specificity. The *false negative rate*, also known as a *Type II error*, is when the screening measure predicts a pass while the older driver actually failed the on-road assessment and is calculated as 1- sensitivity (Streiner and Cairney, 2007; Portney and Watkins, 2009).

Furthermore, *positive predictive value* (PPV) estimates the proportion of older drivers who actually failed the on-road assessment from the total number of older drivers classified as a fail by the screening measure. The *negative predictive value* (NPV) estimates the proportion of older drivers who actually passed the on-road assessment from the total number of older drivers classified as a pass by the screening measure. Values of PPV and NPV that are close 1.00 suggest a higher probability of correctly classifying older drivers into passing/failing categories (Krzanowski and Hand, 2009; Portney and Watkins, 2009).

*Misclassifications* are the number of older drivers that may be erroneously classified by the 32-item FTDS as either passing or failing the on-road assessment. *Error* is the rate of false negative and false positives, represented by the minimum distance between the generated ROC curve and upper left corner of the plot (Krzanowski and Hand, 2009).

The area under the curve (AUC) of the ROC curve represents the screening measure's ability to differentiate between older drivers who passed/failed the on-road assessment (Streiner and Cairney, 2007; Portney and Watkins, 2009). An AUC value that is less than or equal to.50 indicates that the 32-item FTDS is no better than chance at identifying drivers who passed/failed the on-road assessment. Consequently, an AUC value above 0.50 suggests that the 32-item FTDS' can correctly discriminate between drivers that passed/failed the on-road assessment. An AUC between 0.7 and 0.9 indicates moderate accuracy and AUC above 0.9 indicates high accuracy (Fischer et al., 2003).

ROC curve analysis can be used to determine the optimal cut-point or quantifiable score utilized as a criterion for older drivers who passed/failed the on-road assessment. The optimal cut-point is the point where the overall number of misclassifications (false positives + false negatives) is the lowest (Streiner and Cairney, 2007). Visually, the optimal cut-point is the maximum distance between the generated ROC curve and the diagonal line representing an AUC value of 0.50. However, empirically this cut-point can be calculated with Youden's index (*J*). This index ranges from 0 to 1, with values closer to 1 suggesting that the overall effectiveness of a cutoff point is relatively large. Values closer to 0 suggest limited effectiveness (Youden, 1950).

### Data Analysis

A ROC curve was used to summarize the classification success of the measure at the optimal cut-point. Using the formula presented by Hajian-Tilaki (2014), we calculated the minimally acceptable sample size that would allow us to construct a 95% confidence interval with a margin of error that does not exceed 0.10, and a point estimate for sensitivity equal to 0.75. Assuming a prevalence estimate of no less than 0.40, this calculation required a sample size of approximately 181.

In creating this curve, we plotted the true positive rate (sensitivity) against the false positive rate (1-specificity) and then computed the AUC for the ROC. We used an AUC of ≥0.70, *p* < 0.05, as the criterion demonstrating moderate accuracy for predicting older drivers on road-assessment outcomes as an acceptable index of discrimination (Fischer et al., 2003). Then we used the maximum *J* value as the criterion for selecting the optimal cut-point from all possible cut-points (Youden, 1950). Standard error for AUC was computed using Delong's method (DeLong et al., 1988), and this standard error was used to establish confidence intervals around the estimate. Thus, an FTDS score less than the optimal cut-point meant the FTDS predicted the older driver had failed the on-road assessment. Likewise, a mean score greater than, or equal to, the optimal cut-point on the FTDS meant the driver was predicted to pass the on-road assessment. If the AUC did not meet the criterion (≤ 0.70, *p* < 0.05), no further analysis would be carried out. After identifying the optimal cut-point in this fashion, we calculated sensitivity, specificity, PPV, NPV, misclassifications, and error for the groups created. R Statistics software version 3.1.2 (R Core Team, 2015) was used for all analyses, and the ROC curve was fit and analyzed using the pROC package (Robin et al., 2011).

## Results

### Participant Demographics

Two hundred (110 male and 90 female) older drivers and 200 proxy raters (55 male and 145 female) participated in the primary study. On average, the proxy raters were younger (*M* = 62.44 years, *SD* = 14.76) than the older drivers (*M* = 73.64 years, *SD* = 5.35). From the 200 older drivers, 169 passed (84.5%) the on-road assessment, while 31 drivers (15.5%) failed. The descriptive statistics of the driver and proxy rater demographics are published in Classen et al. (2015). Participant mean scores on the 32-item FTDS, as measured on the Likert scale was 4.82, *SD* = 0.27 (range = 3.19–5.00).

### ROC Curve

#### 32-Item FTDS

**Figure 1:**
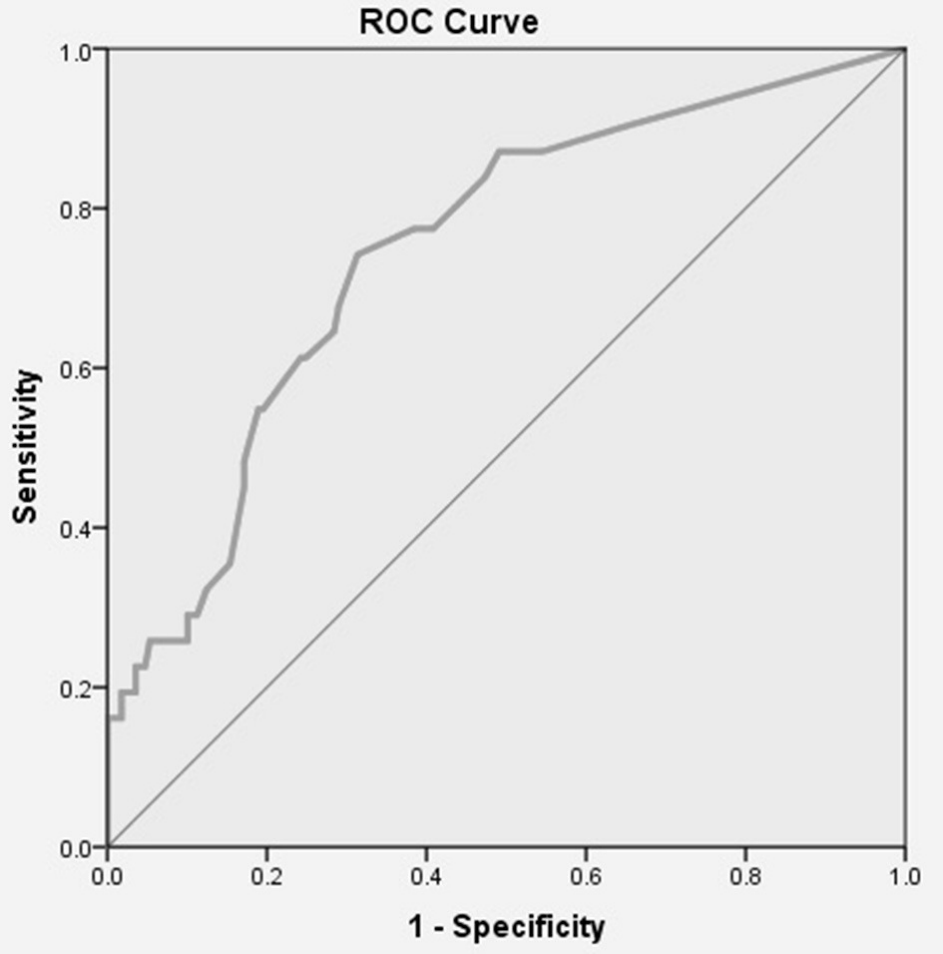
ROC Curve for the 32-item FTDS. AUC = 0.75, *p* < 0.05, 95% CI [0.65, 0.84], *SE* = 0.04.

Figure 1 presents the ROC curve and AUC for the 32-item FTDS. The AUC for the measure was acceptable at a value of 0.75, *p* < 0.05, 95% CI [0.65, 0.84], *SE* = 0.04. Youden's index results indicated that the optimal cut-point was a mean score of 4.87 for driver ability (range = 1–5) on the 32-item FTDS.

Table 1 displays the 32-item FTDS' pass/fail classifications of older drivers based on the optimal cut-point of mean score 4.87 for driver ability on FTDS items. This optimal cut-point yielded a sensitivity of 0.74 and specificity of 0.69. Based on this optimal cut-point, the PPV was 0.30, and the NPV was 0.93. The 32-item FTDS had an error rate of 0.57 and misclassified 61 (out of 200, or 31%) of the older drivers.

**Table 1:** 32-item FTDS' classification of older drivers based on the optimal cut-point of mean score 4.87 for driver ability.

	**On-road assessment outcomes**		
**32-item FTDS outcomes**	**Fail**	**Pass**	**Total**
Fail	23	53	76
Pass	8	116	124
Total	31	169	200

*Sensitivity = 0.74, Specificity = 0.69, PPV = 0.30, NPV = 0.93, Misclassifications = 61/200, Error = 0.57*.

## Discussion

The purpose of this study was to establish the concurrent criterion validity of a shorter FTDS that may need less time to complete than the 54-item FTDS, thereby potentially increasing the utility of the FTDS. Specifically, using existing data and a ROC analysis, this study (1) determined whether the 32-item FTDS predicted pass/fail outcomes of an on-road assessment; (2) established the optimal cut-point for the 32-item FTDS; and (3) quantified the 32-item FTDS' accompanying sensitivity, specificity, PPV, NPV, misclassifications and error rate at the optimal cut-point.

### Participant Demographics

In this study, the proxy rater characteristics were representative of American and Canadian caregiver populations. Similar to caregiver trends in the U.S. and Canada the majority (72.5%) of proxy raters in this study were of the female gender and younger (*M* = 62.44 years) than the older drivers (*M* = 73.64 years) they cared for (Family Caregiver Alliance, 2010). Furthermore, the average age of proxy raters (62.44 years) in the study was comparable to those of caregivers (63 years) examined in the literature (Family Caregiver Alliance, 2010).

### ROC Curve

For the 32-item FTDS, the AUC value indicated that the measure could correctly discriminate between drivers that passed/failed the on-road assessment better than chance. For the 32-item FTDS, the optimal cut-point of mean score 4.87 for driver ability on the FTDS items was the criterion score used to classify older drivers as either passing or failing the on-road assessment. Based on the optimal cut-point the 32-item FTDS had a higher sensitivity than specificity value. Thus, the measure had a higher probability of correctly classifying older drivers who actually failed the on-road assessment as failing, than classifying older drivers who actually passed the on-road assessment as passing. Specifically, the 32-item FTDS is more likely to correctly identify those who have a mean score of less than 4.87 on the FTDS to fail, than those who have a mean score equal to or greater than 4.87 to pass. A higher sensitivity suggests more cases of at-risk older drivers will be identified. A consequence of this may include more drivers receiving the help and resources they need to allow them to stay on the road safer for longer or to consider driving cessation in a timely manner.

Lower specificity at the optimal cut-point suggests there are more false positive (*Type I*) than false negative (*Type II*) errors. That is, out of the 61 misclassifications for the 32-item FTDS, the majority of the older drivers were incorrectly classified as failing (*n* = 53) when they passed the on-road assessment. Consequences of *Type I* error (false positives) may include increased stress, anxiety, and financial hardships due to potentially being classified as unfit to drive. For example, completing a comprehensive driving evaluation can be time-consuming (approximately 3 h to complete), and costly (ranging anywhere from $450 in Florida to over $1000 in New York), often with no third-party reimbursement (Joseph, 2013; American Automobile Association, 2017). As such, these issues may potentially result in unnecessary stress and/or financial hardship for the older driver (Weaver and Bédard, 2012).

Although the on-road assessment is considered to be the gold standard, pass/fail outcomes may be subjective and based on the evaluator's assessment (Larsson and Falkmer, 2007). The background of the evaluator (e.g., driving school instructor vs. certified driver rehabilitation specialist) and protocol being used to assess driving, may impact the objectivity and validity of the outcome, resulting in low reliability (Larsson and Falkmer, 2007). To control for this in the primary study, the driving school instructor was trained by the certified driver rehabilitation specialist on the protocol, and the interrater reliability for on-road assessments between the certified driver rehabilitation specialist (Florida site) and driving school instructor (Ontario site) was assessed. Interrater reliability for the evaluators assessing the same participants' on-road assessment was 100% (Classen et al., 2010).

Only 13% (*n* = 8) of the misclassifications made by the 32-item FTDS were *Type II* errors. That is, drivers were misclassified by the measure as passing the on-road assessment when they actually failed. *Type II* errors can also have negative consequences for the older driver. For instance, older drivers classified as fit to drive, when they are not, may erroneously continue to drive, and as such have a higher risk of being involved in an adverse event (e.g., a crash) due to compromised fitness to drive abilities (Weaver and Bédard, 2012). The relative cost of *Type II* errors (crashes, injuries or fatalities) compared to *Type I* errors (expenses of a driving evaluation, or unnecessary social isolation and emotional stress due to misclassifications) may depend on different factors such as the drivers themselves or location (Weaver and Bédard, 2012). For example, false positive may be more detrimental to individuals living in rural areas than urban cities because urban cities often have alternative forms of transportation available to enable continued community mobility among those who can no longer drive.

The 32-item FTDS had high sensitivity but low PPV. This result may have stemmed from the ratio of those who have passed (169 drivers) vs. failed (31 drivers) the on-road assessment. When dichotomous (e.g., pass/fail) outcomes are very different in number, as in this case, most standard algorithms favor the larger group (pass outcomes) and as such resulting in poorer accuracy in the smaller group's (fail outcomes) predictive value. As a result, spectrum bias may have been introduced (Lin and Chen, 2012; Weaver and Bédard, 2012). Spectrum bias is the phenomenon where the performance (AUC, sensitivity, specificity) of a measure (e.g., FTDS) may change from setting to setting because of the participant sample used (Lin and Chen, 2012). Older drivers who were confident in their driving skills may have been more likely to enroll in this study compared to unconfident drivers, possibly contributing to our sample's disproportionate number of pass vs. fail outcomes. As shown by Myers et al. (2008) the confidence of older drivers is inversely correlated to situational avoidance, as driver's confidence increases, situational avoidance (e.g., driving at night) decreases and vice versa.

The NPV was also higher than the PPV, indicating that 93% of the pass classifications made by the 32-item FTDS were correct whereas only 30% of the fail classifications were correct. This result suggests that positive classifications must be interpreted with caution, as some of these classifications may be false positives. The negative classifications must also be interpreted with caution as some of these classifications may be false negatives. However, the occurrence of false negatives (7%) is less likely to occur than a false positive (70%). The high NPV and low PPV may have also been an artifact of spectrum bias.

The 32-item FTDS demonstrated acceptable concurrent criterion validity with the gold standard on-road assessment, yet, misclassifications (*n* = 61) existed in predicting older driver's fitness to drive. Any misclassification or error when predicting pass/fail outcomes of older drivers can negatively impact older drivers and their loved ones in a multitude of ways, including financially, emotionally and/or in the form of an adverse event including bearing the burdens of a crash, such as cost, injury or death. Despite the presence of error (and misclassification), this study's results suggest that the shorter version of the FTDS yields acceptable validity for further development. Specifically, if this measure is to be used instead of the 54 item FTDS, then a web-based version and a mathematical algorithm for scoring must be developed.

Because participant data were collected between 2008 and 2012, certain vehicle (e.g., advanced driver assistance systems in modern cars) or environmental features (e.g., more recent introductions of roundabouts as traffic calming devices), were not controlled for. Future research should include measurement of the effects of these in-vehicle technologies, as they become more common within standard vehicle equipment lists. Similarly, future research should specifically evaluate road enhancements in FL, U.S., and ON, Canada.

### Implications for Clinicians

Initial validity testing of the measure indicated that the 32-item FTDS developed for proxy raters may be used to screen for and identify at-risk older drivers. Specifically, proxy rater responses to items in the measure can be used to predict older driver pass/fail on-road assessment outcomes. An implication of the 32-item FTDS is that clinicians (such as occupational therapists) may use the results of the measure alongside other clinical information (e.g., client history, collateral information, and results from visual, cognitive and motor tests) to make evidence-informed decisions about the fitness to drive of their clients, but these assertions must be empirically tested.

Implications of the 32-item FTDS for clinicians practice are:

- The 32-item FTDS lays the groundwork for a valid (but shorter) version of the FTDS, but requires further web-based and scoring development.
- The 32-item FTDS sets the stage for a more time efficient measure, but this must be validated empirically.
- The 32-item FTDS may inform clinical reasoning for fitness to drive decisions but this must be tested in a parallel arm design (clinicians using vs. not using the 32-item FTDS for fitness to drive decision-making).

## Conclusion

The results of this study indicate the 32-item FTDS has shown validity (AUC = 0.75, *p* < 0.05) against the gold standard on-road assessment for predicting older driver on-road outcomes. However, due to driver misclassifications the tool must be administered with caution as it may be overly sensitive. Clinicians are advised to use this screening measure, but follow-up assessment for fitness to drive certainty, is recommended.

## Author Contributions

SM performed all analysis, interpreted data, wrote the manuscript. SC supervised development of the work, and assisted with data interpretation and manuscript development. AJ assisted with data analysis and manuscript preparation.

### Conflict of Interest Statement

The authors declare that the research was conducted in the absence of any commercial or financial relationships that could be construed as a potential conflict of interest.
